# JAM-A facilitates hair follicle regeneration in alopecia areata through functioning as ceRNA to protect VCAN expression in dermal papilla cells

**DOI:** 10.1093/pcmedi/pbac020

**Published:** 2022-08-17

**Authors:** Minjuan Wu, Chen Xu, Junfeng Jiang, Sha Xu, Jun Xiong, Xiaoming Fan, Kaihong Ji, Yunpeng Zhao, Haitao Ni, Yue Wang, Houqi Liu, Zhaofan Xia

**Affiliations:** Department of Histology and Embryology, Naval Medical University, Shanghai 200433, China; Burns Institute of People's Liberation Army, Changhai Hospital, Naval Medical University, Shanghai 200433, China; Department of Histology and Embryology, Naval Medical University, Shanghai 200433, China; Spine Center, Department of Orthopedics, Changzheng Hospital, Naval Medical University, Shanghai 200003, China; Department of Histology and Embryology, Naval Medical University, Shanghai 200433, China; Department of Histology and Embryology, Naval Medical University, Shanghai 200433, China; Department of Histology and Embryology, Naval Medical University, Shanghai 200433, China; Burns Institute of People's Liberation Army, Changhai Hospital, Naval Medical University, Shanghai 200433, China; Department of Histology and Embryology, Naval Medical University, Shanghai 200433, China; Department of Histology and Embryology, Naval Medical University, Shanghai 200433, China; Department of Histology and Embryology, Naval Medical University, Shanghai 200433, China; Department of Histology and Embryology, Naval Medical University, Shanghai 200433, China; Translational Medicine Center, Naval Medical University, Shanghai 200433, China; Department of Histology and Embryology, Naval Medical University, Shanghai 200433, China; Burns Institute of People's Liberation Army, Changhai Hospital, Naval Medical University, Shanghai 200433, China

**Keywords:** alopecia areata, dermal papilla cells, JAM-A 3′ UTR, miR-221-3p, VCAN

## Abstract

The dermal papilla cells in hair follicles function as critical regulators of hair growth. In particular, alopecia areata (AA) is closely related to the malfunctioning of the human dermal papilla cells (hDPCs). Thus, identifying the regulatory mechanism of hDPCs is important in inducing hair follicle (HF) regeneration in AA patients. Recently, growing evidence has indicated that 3′ untranslated regions (3′ UTR) of key genes may participate in the regulatory circuitry underlying cell differentiation and diseases through a so-called competing endogenous mechanism, but none have been reported in HF regeneration. Here, we demonstrate that the 3′ UTR of junctional adhesion molecule A (JAM-A) could act as an essential competing endogenous RNA to maintain hDPCs function and promote HF regeneration in AA. We showed that the 3′ UTR of JAM-A shares many microRNA (miRNA) response elements, especially miR-221–3p, with versican (VCAN) mRNA, and JAM-A 3′ UTR could directly modulate the miRNA-mediated suppression of VCAN in self-renewing hDPCs. Furthermore, upregulated VCAN can in turn promote the expression level of JAM-A. Overall, we propose that JAM-A 3′ UTR forms a feedback loop with VCAN and miR-221–3p to regulate hDPC maintenance, proliferation, and differentiation, which may lead to developing new therapies for hair loss.

## Introduction

Alopecia areata (AA) is a non-scarring, autoimmune hair loss disorder characterized by the atrophy and upper shift of the hair follicles (HFs), bulb, and human dermal papilla cells (hDPCs), with lymphocyte infiltration.^1–3^ Dermal papilla (DP) cells are recognized as the key inductive mesenchymal player.^4^ HFs are self-regenerating miniorgans that form in recurring cycles, beginning with their embryonic development.^5^ hDPCs agglomerate into bulbous cavities that serve as the hub of a signaling network that directs the activation of epithelial progenitors and regularly initiates the regeneration process for HFs over the course of all hair growth developmental stages.^6,7^ Thus, hDPCs and HFs can be used as highly tractable and well-understood model systems for exploration of numerous unresolved biological questions, such as those involving cell-to-cell interactions and signaling and cell behavior during development.

In addition to their relevance for basic biological questions, the study of hDPCs can lead to clinically applied findings for treatment of issues surrounding hair loss or excessive hair growth (hirsutism, hypertrichosis). Previous research has shown that hair loss is induced by apoptosis and dysfunction of hDPCs.^8,9^ Advances in characterization of the genetic and biochemical regulatory mechanisms governing the cyclic regeneration or dysfunction of hDPCs can potentially lead to promising therapies for hair loss as well as HF-based engineered tissues.

Among their many important characteristics, the agglutinative growth of hDPCs is directly related to its pluripotency as differentiating stem cells,^10^ specifically, hair inductivity. The agglutinative growth of DPCs in this paper is defined as DPCs gathering in clusters when growing. Several reports have shown that following a fifth passage, hDPCs in culture lose both their ability to coagulate as well as their hair inductivity.^11,12^ Furthermore, the expression of marker proteins including alkaline phosphatase (ALP),^13^ and SOX-2^14^ are also decreased in hDPCs. In-depth studies into the regulatory mechanisms governing hDPCs agglutinative growth have shown that VCAN (versican, a chondroitin sulfate proteoglycan matrix core protein) is an essential component in the processes of mesenchymal condensation and induction of hair growth.^12,15^ Although VCAN has been shown to participate in the transformation of HFs from the regressive to the growth phase,^16^ the mechanisms by which VCAN and changes in its expression regulate hDPCs maintenance remain largely unexplored.^17,18^

As a type of adhesion molecule, junctional adhesion molecule A (JAM-A), also known as JAM-1 or F11R, belongs to the immunoglobulin protein superfamily. JAM-A is widely expressed in various cells and is known to play a critical role in fostering vascularization.^2,19,20^ Our prior study demonstrated that knockdown of JAM-A resulted in inhibition of mesenchymal stem cells' (MSCs) migration ability.^21^ In this study, we demonstrate that the functions of JAM-A mRNA are dependent on its 3′ untranslated region (UTR), which is a competitive endogenous RNA (ceRNA) that competes to regulate VCAN expression. This work provides considerable insight into the roles and interactions of adhesion-related molecules in HF regeneration, which can advance our basic understanding of these proteins in stem cells differentiation in other tissues, as well as serving as potentially strong therapeutic targets in hair loss disorders.

## Materials and methods

### Isolation, cultivation, and characterization of hDPCs

hDPCs were isolated from abandoned human scalp tissue and placed in the bottom of a 60-mm dish for culture in Dulbecco's Modified Eagle Medium (DMEM) (Invitrogen, Carlsbad, CA, USA) containing 1% penicillin and streptomycin and 10% fetal bovine serum (FBS) (Gibco, Gaithersburg, MD, USA) at 37°C under 5% CO_2_, following previously reported protocols.^22,23^ When confluent, primary cultured cells were harvested and re-seeded (2.0 × 10^4^ cells/dish), then subsequently passaged on a weekly basis.

### hDPCs transfection

hDPCs were trypsinized and seeded at 2 × 10^4^ cells in 1 ml of DMEM supplemented with 10% FBS in a 12-well plate. Then, 10 μl of virus solution was added in the presence of 5 μg/ml Polybrene (Shanghai Genechem Ltd, Shanghai, China). Lentiviral vectors were used to overexpress or knockdown JAM-A and VCAN in hDPCs (Shanghai Genechem Ltd, Shanghai, China). The siRNA sense and antisense oligo nucleotides complementary to human JAM-A and VCAN mRNA were designed following previously published methods.^24^

For studies of miRNA function and gene regulation, miRNA mimics and inhibitors of miR-221–3p^25^ were also purchased from a commercial vendor (Genepharm, Inc., Sunnyvale, CA, USA), and the sequences of all siRNAs, miRNA mimics, and miRNA inhibitors are listed in Supplementary Table S1. hDPCs were transfected with 20 nM miRNA mimic or 20 nM miRNA inhibitor according to a previously described method for siRNA transfection of hDPCs.^26^ Fugene HD reagent (Promega, Madison, WI, USA) or lipofectamine RNAi MAX (Invitrogen, Carlsbad, CA, USA) was used for vector or RNA oligos transfection, respectively, according to the manufacturer's instructions. At the time of transfection, the optimal confluency for hDPCs is 60%–70%.

### RT–qPCR relative expression analysis of mRNA and microRNA

Total RNA was extracted using Trizol reagent (ThermoFisher Scientific, Waltham, MA, USA). RNA isolation, cDNA synthesis, and PCR amplification were performed as previously described.^26^ For relative mRNA expression analysis, RT–qPCR was carried out on a Step One plus System (Applied Biosystems, Waltham, MA, USA), using a Power SYBR Green PCR Master Mix (Applied Biosystems, Waltham, MA, USA). The primers for JAM-A, JAM-A 3′ UTR, VCAN, and the β-actin internal reference housekeeping gene are listed in Supplementary Table S2.

The expression of select miRNAs was also quantified using the Step One plus System, which converts miRNAs into cDNA, then detects copy number using SYBR Green-based real time PCR. The primer sequence for miRNA amplification is given in Supplementary Table S3.

### Cell cycle analysis

For cell cycle analysis, about 1 × 10^6^ treated or untreated hDPCs were collected and fixed with 80% ice-cold ethanol for 2 hours. After washing and centrifugation, 0.5 ml of PI/RNase (BD Pharmingen, San Diego, CA, USA) was added to the cell lysis and incubated at room temperature for 15 min in the dark. After trypsin treatment, cells were resuspended by pipetting, 1 ml of complete culture media was added and 500 μl of the suspension was transferred into FACS tubes.^26^ The flow cytometry data were analyzed using ModFit LT 5.0 software (Verity Software House, Topsham, ME, USA).

### CCK-8 assays

The Cell Counting Kit-8 (CCK-8) (Toyobo, Japan) was also used to evaluate hDPC proliferation. In accordance with the manufacturer's protocol, all DP cells were seeded in 96-well plates at 2 × 10^3^ cells/well (five wells in each group), cultured in DMEM supplemented with 10% FBS. Ten microliters of the CCK-8 solution was added to each well, and the plate was incubated at 37°C for 1 h. The optical density was then read at 450 nm using a microplate reader (Tecan, Austria).

### Luciferase reporter transfection and dual luciferase assay

Luciferase reporter transfection and dual luciferase assays were performed following protocols established in our previous reports.^26,27^ We used the computational target prediction algorithm miRanda (http://cbio.mskcc.org/microRNA2003/miranda.html) to identify mature gene transcripts that were potentially targeted by JAM-A at their 3′ UTRs. VCAN was then experimentally verified as a JAM-A target gene in hDPCs following the protocols of Wang *et al*.^26,27^ Reporter assays used Light Switch 3′ UTR Go Clone Constructs (BioCat GmBH, Heidelberg, Germany) based on the 3′ UTR sequences of JAM-A and VCAN, which were co-transfected with 100 ng of VCAN reporter plasmid and either 10–40 nM miRNA-221–3p mimic or 40 nM control siRNA. After 24 h of incubation, 50 μl of Light Switch reagent (BioCat GmBH, Heidelberg, Germany) was added to each well, and luminescence was measured using a plate-reading luminometer (Tecan, Männedorf, Switzerland). RT–qPCR analysis was performed to quantify the VCAN transcription levels in DP cells at 1 day after incubation with the microRNA-221–3p mimic or inhibitor.

### Intracutaneous injection of hDPCs

Engraftment was initiated by intracutaneous injection of 2 × 10^4^ hDPCs to the forelimb dorsal skin of 21-day-old BALB/c-nu/nu mice. Prior to injection, cells were suspended in 150 μl of saline; equivalent volumes of saline were injected as a negative control. Histological analysis of male BALB/c-nu/nu mouse skin was performed by staining with hematoxylin and eosin (Richard Allan Scientific, San Diego, CA, USA). We macroscopically observed hair growth at the implantation area every day following the injection.

### Analysis of *in vivo* hair regeneration

To observe the relative capacity for initiation of hair regeneration in the skin of male BALB/c-nu/nu mice, skin tissue was removed and placed in 30% sucrose for 24 hours. The tissues were then embedded in Tissue-Tek OCT (Sakura Finetek USA, Torrance, CA, USA) and sectioned into 8-μm cryostat sections.

### AA model mouse preparation and JAM-A-3′ UTR transfection

Imiquimod was employed to induce 6-week-old C3H/HeJ mice to form AA. Imiquimod ointment applied once or twice daily for 4 weeks is more efficacious to induce AA regeneration in C3H/HeJ mice.^28–30^ To assess the severity of hair loss or AA, H&E staining was used to detect whether the model of AA is successfully constructed. AA were transduced with a vector or JAM-A-3′ UTR lentiviral vector, miR-221, or inhibitor lentiviral vector (Shanghai Genechem Ltd, Shanghai, China).

Lentivirus packaging system and human embryonic kidney-293T (HEK-293T) cells were used to prepare the lentivirus overexpressing JAM-A-3′ UTR. The recombinant virus preparation was titered, and the expression of JAM-A-3′ UTR in C3H/HeJ mice was confirmed by frozen section. Hair regrowth was monitored through gross observation.

### Immunofluorescence and FISH

These assays were performed following protocols in previously published reports.^26,31^ The subcellular localization of JAM-A, VCAN, and ALP proteins in the hDPCs was examined by immunocytochemistry, as previously described.^31^ Culture slides were incubated with rabbit anti-JAM-A (ab180821, Abcam), rabbit anti-VCAN (ab202906, Abcam), and mouse anti-ALP antibodies (ab65834, Abcam) at a 1:100 dilution in a solution containing 0.5% Triton X-100 and 1% bovine serum albumin (BSA) for 2 h at 37°C. After several washes in phosphate buffered saline (PBS), the culture slides were incubated with rhodamine-conjugated anti-goat IgG-R, fluorescein-conjugated anti-mouse IgG-R (Invitrogen, Carlsbad, CA, USA) at a 1:500 dilution for 45 min at room temperature and visualized using an Olympus IX70 microscope (Olympus Corporation, Shinjuku, Tokyo, Japan).

For the detection of JAM-A and VCAN transcripts, samples were further hybridized with FISH probe reaction buffer-1 (Genepharm Inc., Sunnyvale, CA, USA) containing respective RNA probes. RNA probes were labelled with 5′ CY3 (Genepharm, Inc., Sunnyvale, CA, USA). For observation of tissue samples, mouse anti-mitochondria (1:100, ab3298, Abcam), rhodamine-conjugated anti-mouse IgG-R, and fluorescein-conjugated anti-rabbit IgG-R antibodies were used. Nuclei were fluorescently labeled with DAPI (Invitrogen, Carlsbad, CA, USA). All secondary antibodies were purchased from Invitrogen.

### Western blot

Protein extraction from the hDPCs, SDS–polyacrylamide gel electrophoresis, and western blotting were performed as previously described.^32^

### RNA immunoprecipitation (RIP) assay

The MS2bp-MS2bs-based RIP assay was performed according to previously established protocols,^26,33^ with modifications for using the EZ-Magna RIP Kit (Millipore Sigma, Burlington, MA, USA) in accordance with the manufacturer's instructions.

### Statistical analysis

Data are presented as means ± SD. For data from RT–qPCR, western blots, and luciferase activity assays, differences between groups were assessed by a paired, two-tailed Student's *t*-test. *P* < 0.01 was used to determine statistical significance.

## Results

### Clustering analysis reveals differentially expressed adhesion-related genes in AA and normal scalp tissues

**Figure 1.**
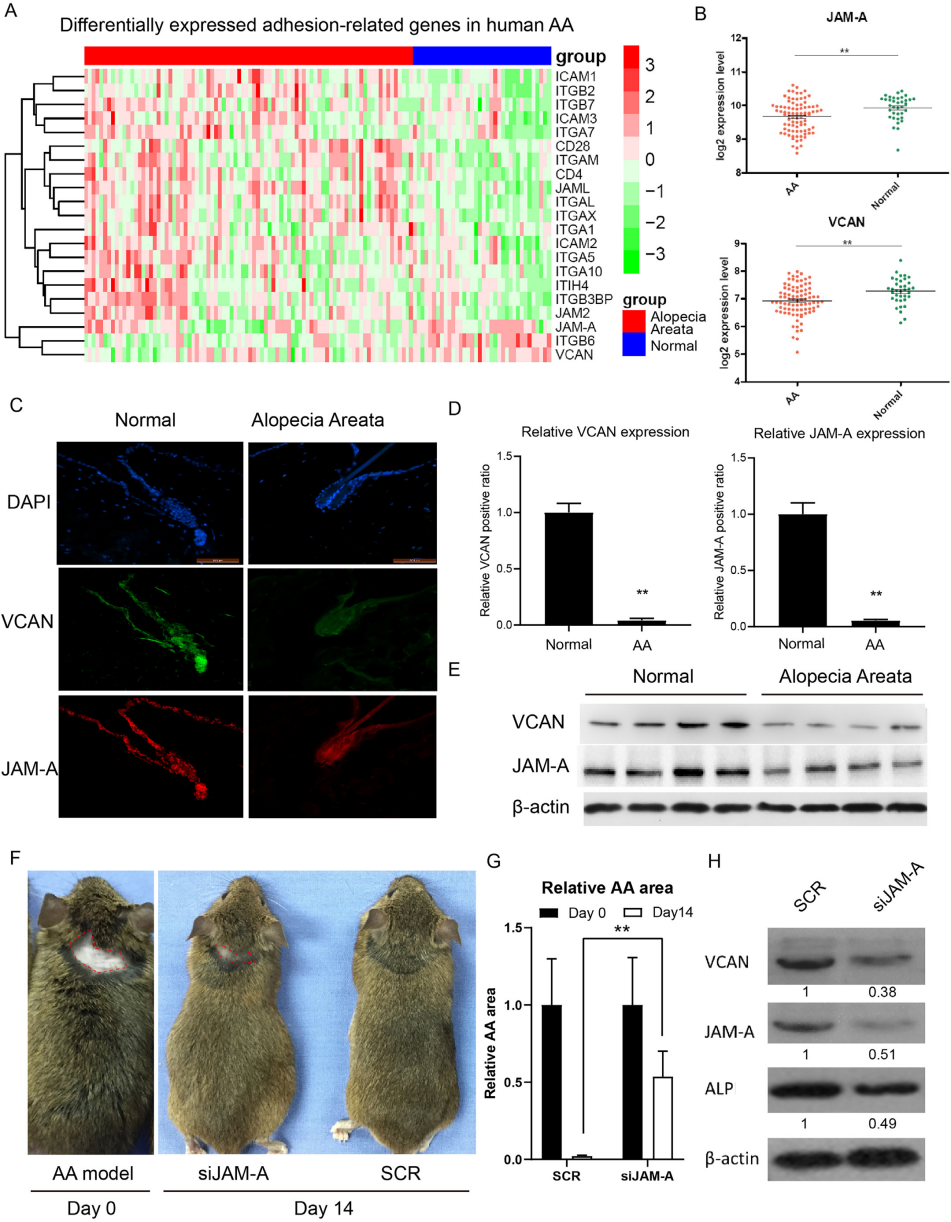
JAM-A and VCAN are down regulated in AA. (**A**) Heat map of differentially expressed adhesion-related genes with green indicating down-regulation of genes and red indicating up-regulation of genes (top bar: blue samples are scalp skin from normal tissue; red samples are scalp skin from patients with AA). Data Sources GSE68801. (**B**) Compared with healthy people, the relative expression levels of JAM-A and VCAN in the skin and scalp samples of patients with AA were lower. (**C**) The results of hematoxylin and eosin staining showed that in the scalp tissue of the control group, the hair follicles penetrated deep into the fat cells of the subcutaneous tissue, while in the scalp of patients with AA the hair follicle was shorter in length and confined to the dermis tissue. The results of immunohistochemistry showed that VCAN and JAM-A were enriched in the papilla of hair follicles, which was brown. (**D**) Relative expression levels of JAM-A and VCAN in AA patient's tissue were lower compared to normal samples. (**E**) Western blot analysis showed that the relative expression level of JAM-A and VCAN in the scalp of patients with AA was lower than that of normal people. (**F**) We downregulated JAM-A expression in AA model mice (left) (subcutaneous injection JAM-A shRNA expressing lentivirus). After 14 days, we observed the hair regrowth of AA was delayed in siJAM-A group mice. (**G**) Quantification of germinal area. In the JAM-A interference group, the number and speed of hair growth were lower than those in the SCR group. (**H**) Western blot analysis showed that the relative expression level of JAM-A, AP, and VCAN in JAM-Akd group was lower than that of SCR group. Three repetitions, data are represented as means ± SD. *P < 0.05, **P < 0.01.

To identify differences in gene expression patterns related to AA, we performed two independent microarrays and clustering analysis on several adhesion-related genes. Through this relative expression analysis (patients with AA and healthy scalp, Data Sources GSE68801), we found that JAM-A, VCAN, and ITGB6 were down-regulated in scalp tissue of patients with AA (Fig. 1A). We also found that the relative expression levels of both VCAN and JAM-A in AA patients were significantly lower compared to normal patients (Fig. 1B). This finding strongly suggested that both JAM-A and VCAN expression were related to hair baldness.

Histological analysis revealed the expression of VCAN and JAM-A in the normal HFs (Fig. 1C). Dermal papilla caught our attention. Quantitative real-time PCR (qPCR) and western blot analyses of four samples taken from cases of human AA and four from normal patients confirmed that JAM-A and VCAN were down-regulated in human AA scalps (Fig. 1D, E). JAM-A RNA-interference (siJAM-A) experiments revealed that down-regulation JAM-A expression resulted in the inhibition of hair regeneration (Fig. 1F, G). In addition, ALP and VCAN expression in hDPCs were also diminished in the siJAM-A group (Fig. 1H).

These results strongly suggest that decreased JAM-A and VCAN expression is associated with AA in particular. Given that JAM-A expression is closely related with VCAN expression, ALP expression and hair regeneration, the question remains as to how JAM-A functions mechanistically in these processes. We therefore endeavored to resolve the relationship between JAM-A, VCAN, and hair regeneration.

RT–PCR and western blot results showed that JAM-A and VCAN gene expression changed synchronously in hDPCs (Supplementary Fig. S1). Notably, the expression of JAM-A rapidly decreased in p7 hDPCs, which was simultaneous with the decline in levels of VCAN. We therefore hypothesized that JAM-A might regulate VCAN expression in hDPCs, suggesting a role for JAM-A in the regulatory network governing hDPCs activity and level of differentiation or primitiveness.

### JAM-A 3′ UTR expression is positively correlated with the aggregative behavior of DP cells in vitro

**Figure 2.**
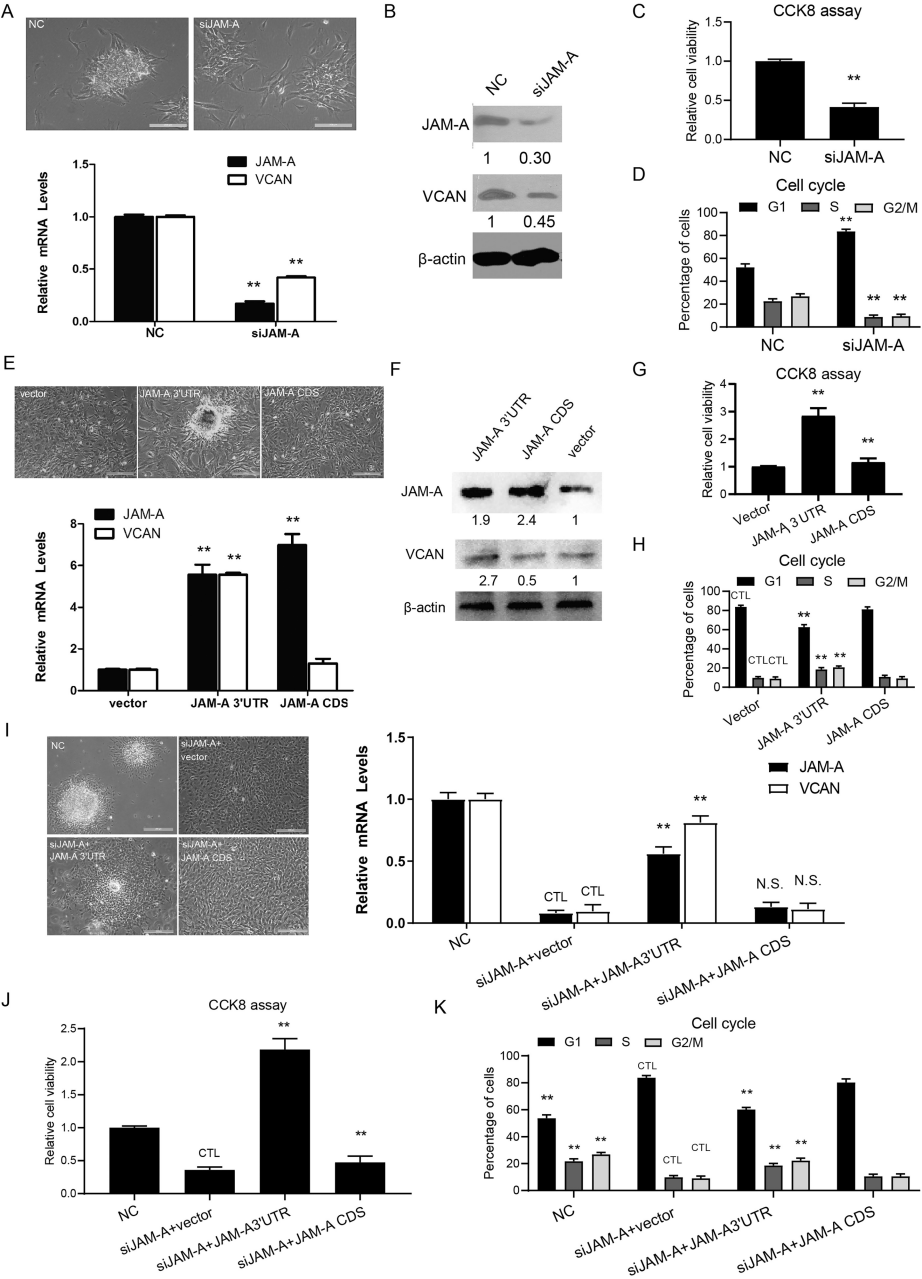
JAM-A promotes agglutinative growth and VCAN expression of hDPCs through the 3′ UTR region. (**A**) Quantitative analysis of JAM-A and VCAN expression in JAM-A knockdown hDPCs, the phenotypic agglutinative colonies were lost in the siJAM-A group. NC, transfected with a scrambled siRNA negative control. (**B**) Western blot analysis revealed the expression of JAM-A and VCAN were decreased in the siJAM-A group compared to NC. (**C**) CCK-8 assays were conducted to determine the cell proliferation. (**D**) Cell viability was assessed by FCM in hDPCs with and without JAM-A knockdown. (**E**) Quantitative analysis of JAM-A and VCAN expression in JAM-A overexpressed hDPCs, the phenotypic agglutinative colonies were shown in the upper panel. The JAM-A CDS represents overexpression of only JAM-A coding region, while JAM-A 3′ UTR represents overexpression of JAM-A 3′ UTR region only. (**F**) Western blot analysis showing the protein expression of JAM-A and VCAN in JAM-A overexpressed hDPCs. (**G**) CCK-8 assays were conducted to determine the cell proliferation of hDPCs with JAM-A CDS or 3′ UTR overexpression. (**H**) Cell viability was assessed by FCM. (**I**) Quantitative analysis of JAM-A and VCAN expression in hDPCs of different treatments, the phenotypic agglutinative colonies were shown in the left panel. The JAM-A CDS represents overexpression of only the JAM-A coding region, while JAM-A 3′ UTR represents overexpression of only the JAM-A 3′ UTR region. (**J**) CCK-8 assays were conducted to determine the cell proliferation of hDPCs with of different treatments. (**K**) Cell viability analysis of hDPCs with different treatments by FCM. All quantifications were done with three independent repeats, and the expression of GAPDH was used as PCR internal references. CTL means control. Data are represented as means ± SD. *P < 0.05, **P < 0.01. Scale bars show 50 μm.

To better understand the individual roles of VCAN and JAM-A^24^ in hDPCs aggregation, we used siRNAs targeting VCAN or JAM-A to posttranscriptionally suppress their expression. Agglutinative growth was also diminished in both VCAN and JAM-A knockdown hDPCs (Fig. 2A, Supplementary Fig. S2). Furthermore, the optimum reduction in the level of VCAN mRNA and protein was detected by RT–qPCR and western blot (Fig. 2A, B). These findings are consistent with previous reports,^32^ suggesting that the JAM-A gene has a close and interdependent relationship with VCAN, and that hDPCs depend on the expression of both genes for characteristics such as agglutinative growth.

**Figure 3.**
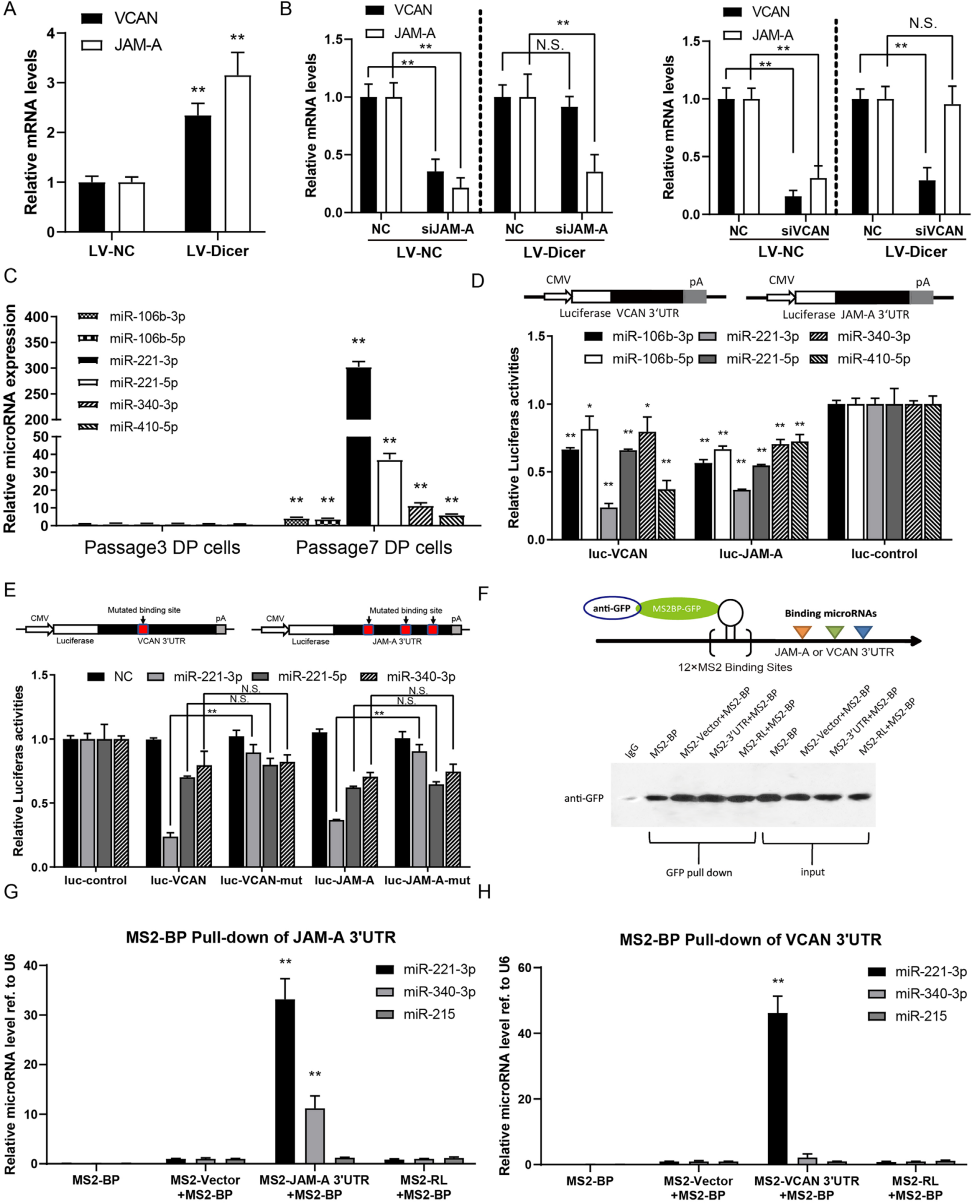
JAM-A 3′ UTR regulates VCAN expression by sequester microRNAs in hDPCs. (**A** and **B**) Quantitative analysis of JAM-A and VCAN expression in lentiviral mediated Dicer knockdown hDPCs with and without JAM-A and VCAN knockdown. (**C**) The Quantification of JAM-A and VCAN targeted miRNAs between early and late passage hDPCs. (**D**) Dual-luciferase assay detecting the targeting effect of candidate miRNAs to VCAN and JAM-A 3′ UTR. (**E**) Dual-luciferase assay detecting the targeting effect of candidate miRNAs to site mutated VCAN and JAM-A 3′ UTR constructs. (**F**) Illustration of the mechanism of RNA immunoprecipitation using the MS2 system. The western blot analysis showing the efficacy of MS2 mediated 3′ UTR pulldown in hDPCs is showing in the lower panel. (**G**) The quantification of microRNAs in MS2 mediated JAM-A 3′ UTR pulldown of hDPCs. (**H**) The quantification of microRNAs in MS2 mediated VCAN 3′ UTR pulldown of hDPCs. All quantifications were done with three independent repeats, and the expression of GAPDH was used as a PCR internal references. Data are represented as means ± SD. *P < 0.05, **P < 0.01.

As shown in Fig. 3C the relative cell viability was decreased by 52% after the treatment of JAM-A interference. The percentage of hDPCs in G1 phase was increased after JAM-A interference (*P* < 0.01), but the proportion of cells in S phase and G2 phase was decreased and the cell cycle was arrested at G1 phase (*P* < 0.01) (Fig. 2D).

To better understand the contribution of JAM-A expression specifically in the process of hDPCs differentiation, we separately examined the effects of JAM-A 3′ UTR and JAM-A CDS (coding sequence) overexpression in maintaining hDPCs aggregation prior to differentiation. Overexpression of the JAM-A 3′ UTR in WT p7 hDPCs restored VCAN expression and agglutinative growth (Fig. 2E). However, overexpression of the full JAM-A CDS in hDPCs at p7 resulted in neither agglutinative growth nor VCAN expression (Fig. 2F), suggesting that expression of the JAM-A 3′ UTR is necessary for hDPCs aggregation and VCAN expression. Meanwhile, the relative cell viability were increased in JAM-A 3′ UTR and JAM-A CDS overexpression (Fig. 2G). FCM analysis demonstrated that overexpression of JAM-A 3′ UTR could decrease the cell proportion of S and G2/M phase in DP cells (Fig. 2H).

As discussed before, we observed the loss of the agglutinative growth phenotype in p3 hDPCs following siRNA-mediated JAM-A depletion, so we overexpressed the JAM-A 3′ UTR or the JAM-A CDS in p3 hDPCs with JAM-A silence (Fig. 2I). The results showed that overexpression of the JAM-A 3′ UTR partially rescued agglutinative growth and VCAN expression, while overexpression of the over expression CDS in siJAM-A hDPCs did not increase VCAN expression or hDPCs aggregation (Fig. 2I). Compared to siJAM-A + vector hDPCs, the relative cell viability were increased in siJAM-A + JAM-A 3′ UTR and siJAM-A + JAM-A CDS (Fig. 2J), the decrease cell proportion of G1 phase, the increase of S and G2/M phase were detected (Fig. 2K).

These results further illustrate the critical role of the JAM-A 3′ UTR in VCAN expression in hDPCs. Considering the close relationship between VCAN and the agglutinative growth phenotype of hDPCs, we therefore propose that the JAM-A 3′ UTR forms a regulator in hDPCs that mediates agglutinative growth and subsequently induces hair regeneration.

### JAM-A 3′ UTR regulates expression of VCAN through a miRNA-dependent mechanism in hDPCs, specifically, JAM-A 3′ UTR shares regulatory miRNAs with VCAN and prevents miRNA-221-3p-mediated VCAN degradation

To further study how the JAM-A 3′ UTR regulates hDPCs aggregation through VCAN, we next evaluated the molecular mechanism underlying JAM-A-mediated regulation of VCAN expression. We evaluated the expression of VCAN in dicer-deficient hDPCs, which were impaired for global expression of miRNAs.^34^ The lentivirus (LV)-short hairpin (sh) RNA-mediated knockdown of dicer mRNA expression in hDPCs was confirmed. In both the undifferentiated p3 hDPCs and differentiated p7 hDPCs, we found that dicer knockdown partially rescued the decrease in VCAN expression caused by JAM-A knockdown, whereas the lentivirus control-infected cells exhibited changes similar to cells infected with the scrambled vector construct (Fig. 3A, B, Supplementary Fig. S3A, B). These results support our hypothesis that the expression of miRNAs is essential for JAM-A 3′ UTR-mediated regulation of VCAN expression. As one of the most important posttranscriptional modifiers, miRNAs have been shown to be critical regulatory factors during hDPCs proliferation and differentiation.^35^

We used the bioinformatic tool miRanda^36^ to search for miRNAs that target the full-length JAM-A, VCAN, BMP-4, β-catenin, and Shh, all of which participate in hair regeneration. Of the miRNAs that fit these criteria, miR-221–3p, miR-221–5p, miR-410–5p, miR-340–3p, miR-106b-3p, and miR-106b-5p emerged as obvious candidates because their predicted binding sites were shared by the JAM-A 3′ UTR and VCAN 3′ UTR (Table S4). These miRNAs were reported to regulate hDPCs’ self-renewal and activity.^25^ The Quantification of JAM-A and VCAN targeted miRNAs were increased in late passage hDPCs (Fig. 3C). For the primer sequence, see Supplementary Table 5.

For further confirmation of the role of miRNAs in regulating hDPCs function, we also created luciferase reporter fusion constructs containing the JAM-A 3′ UTR or the VCAN 3′ UTR and co-transformed hDPCs with either of the 3′ UTR reporter constructs and the individual mimics of the miRNAs identified by miRanda. We found that miR-221–3p mimics substantially reduced the luciferase activities of the reporter vectors containing JAM-A 3′ UTR or VCAN 3′ UTR in HEK-293 cells (Fig. 3D). JAM-A 3′ UTR or VCAN 3′ UTR with miR-221–3p binding site mutation had been constructed. The results of dual luciferase experiments showed that miR-221–3p affected luciferase activities of JAM-A 3′ UTR or VCAN 3′ UTR (Fig. 3E). We therefore chose miR-221–3p as a model miRNA for further study. To validate the direct binding of the predicted miRNA response elements to the transcripts, we then performed an RNA immunoprecipitation (RIP) assay with MS2 binding sequences (MS2bs). We found that RIP pull-downs of endogenous miRNAs associated with the JAM-A 3′ UTR were significantly enriched for miR-221–3p, compared with the empty vector (M2S), and non-targeting miRNA miR-215 (Fig. 3F). We confirmed the enrichment for these miRNAs in hDPCs by RT–qPCR (Fig. 3G, H).

**Figure 4.**
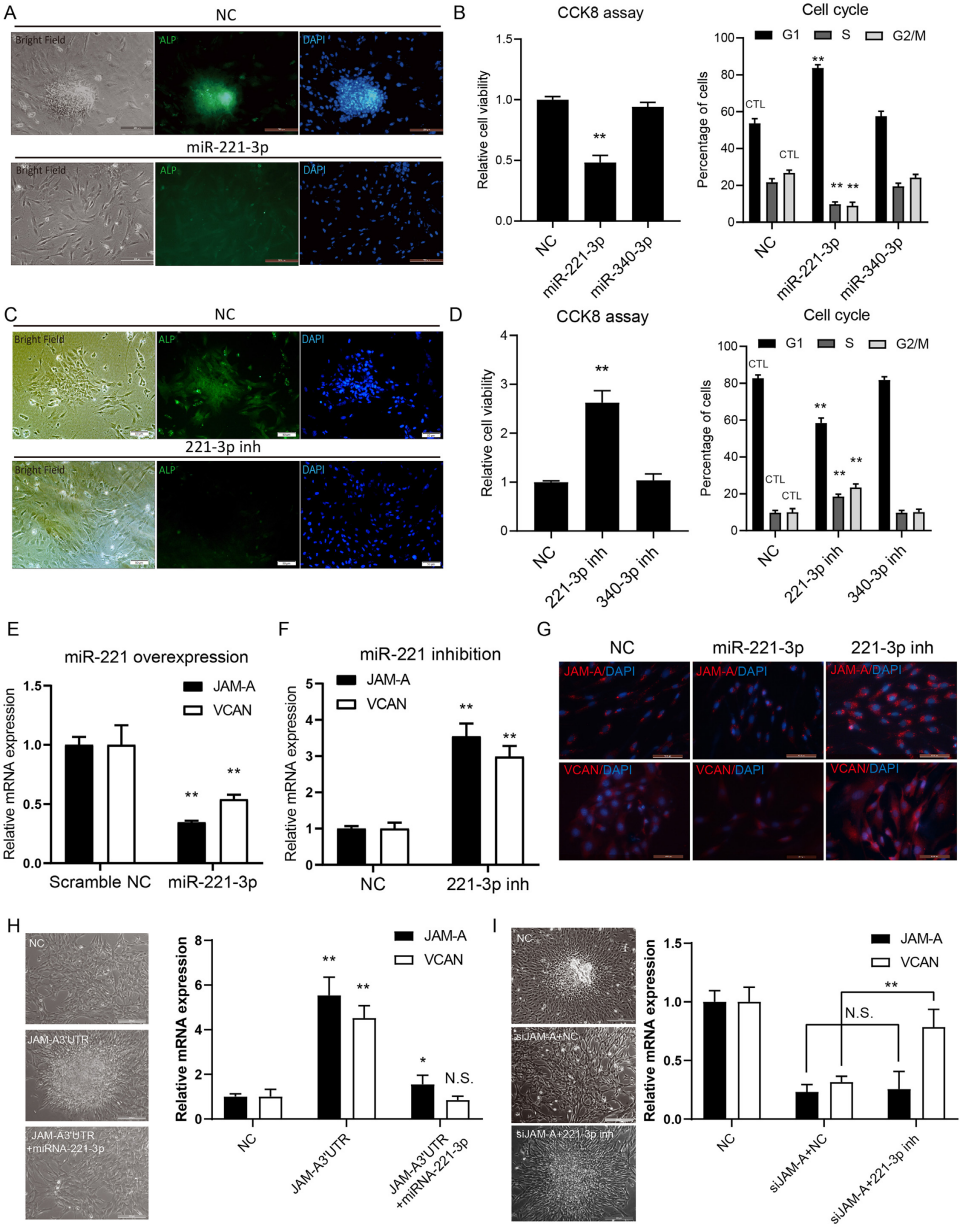
miR-221–3p is necessary for JAM-A 3′ UTR to promote VCAN expression. (**A**) Representative immunofluorescence imaging of AP expression in miR-221–3p transfected hDPCs. (**B**) CCK-8 assays were conducted to determine the cell proliferation of hDPCs with miR-221–3p transfection. The right panel shows the cell viability analysis of miR-221–3p transfected hDPCs. (**C**) Representative immunofluorescence imaging of AP expression in miR-221–3p inhibitor transfected hDPCs. (**D**) CCK-8 assays and FCM were used to observe the effects of miR-221–3p expression on the proliferation and cell cycle of hDPCs. (**E** and **F**) Quantitative analysis of JAM-A and VCAN expression in miR-221–3p mimics and inhibitor transfected hDPCs. (**G**) The immunofluorescence imaging of JAM-A and VCAN expression in miR-221–3p overexpressed or inhibited hDPCs. (**H** and **I**) Quantitative analysis of JAM-A and VCAN expression in miR-221–3p overexpression (**H**) or inhibition (**I**) treated hDPCs in different groups. The agglutinative properties of treated hDPCs were shown using bright field microscopy in the left panel. All quantifications were done with three independent repeats, and the expression of GAPDH was used as PCR internal references. Data are represented as means ± SD. *P < 0.05, **P < 0.01. Scale bars show 50 μm.

To validate the effects of miR-221–3p on preventing the differentiation and aggregation of hDPCs, we transfected the miR-221–3p mimics and negative control RNA (NC) into p3 hDPCs (Fig. 4A). After 5 days of growth, the agglutinative growth phenotype was diminished, and at 7 days after transfection the ability of cells to aggregate was lost completely (Fig. 4A). The expression of alkaline phosphatase (ALP), a marker for progenitor hDPCs, was down-regulated in cells transfected with miR-221–3p (Fig. 4A). Compared to the NC group, the relative cell viability was decreased in miR-221–3p up-regulation hDPCs, the cell proportion of G1 phase were increased (Fig. 4B). Concurrent with miR-221–3p up-regulation, the expressions of VCAN and JAM-A were down-regulated in miR-221–3p-transfected p3 hDPCs (Fig. 4A, E, G). However, in p7 hDPCs exposed to miR-221–3p inhibitor, JAM-A and VCAN transcriptions were restored to levels 3-fold greater than in the controls not treated with inhibitor (Fig. 4C, F, and G). Compared to NC group, the relative cell viability was increased in miR-221–3p inhibitor transfection hDPCs, the cell proportion of G1 phase was decreased (Fig. 4B).

In JAM-A 3′ UTR-overexpressing p7 hDPCs transfected with miR-221–3p mimics, we found that the expression of JAM-A and VCAN were decreased compared to JAM-A 3′ UTR-overexpression group (Fig. 4H). Furthermore, to determine the role of miR-221–3p in the JAM-A 3′ UTR-mediated regulatory loop, we further used the miR-221–3p inhibitor to abolish miR-221–3p up-regulation in p3 hDPCs with JAM-A interference (siJAM-A) (Fig. 4I). The results showed that the expression of VCAN in siJAM-A + miR-221–3p inhibitor hDPCs was increased compared to siJAM-A + NC. Meanwhile, the aggregation of hDPCs partially appeared (Fig. 4I). JAM-A 3′ UTR is just like a suppressor that inhibits hDPCs differentiation and aging. In light of these findings, we speculated that mutation of the miRNA-221–3p binding sites in the JAM-A 3′ UTR would effectively prevent regulation of hDPC differentiation and induce hair regeneration.

To determine whether miR-221–3p directly bound the JAM-A 3′ UTR, we constructed JAM-A 3′ UTR and VCAN 3′ UTR transcript variants with mutations (mut1, 2, 3 and all mut) in putative miR-221–3p binding sites (Supplementary Fig. S4A, B, Table S4). We found that these mutations partially abolished the previously observed inhibitory effects of miR-221–3p on transcription of wild-type JAM-A and VCAN transcription in HEK293 cells (Supplementary Fig. S4).

These data support the hypothesis that in hDPCs proliferation and differentiation, JAM-A 3′ UTR functions as an endogenous miR-221–3p sponge to mitigate VCAN degradation induced by elevated levels of miR-221–3p.

### JAM-A 3′ UTR expression is positively correlated with hair regeneration *in vivo*

To determine whether the JAM-A 3′ UTR has similar effects *in vivo*, we intradermally injected hDPCs with JAM-A over expression (OE) or knockdown (KD) into shoulder skin of 21-day-old nude mice, which was in the first telogen phase of the hair growth cycle.^21,37^ The specific groups are as follows: saline group, hDPCs^scr^, hDPCs^siJAM-A^, hDPCs^siJAM-A+vec^, hDPCs^siJAM-A+UTR^, and hDPCs^siJAM-A+UTR mut^.

**Figure 5.**
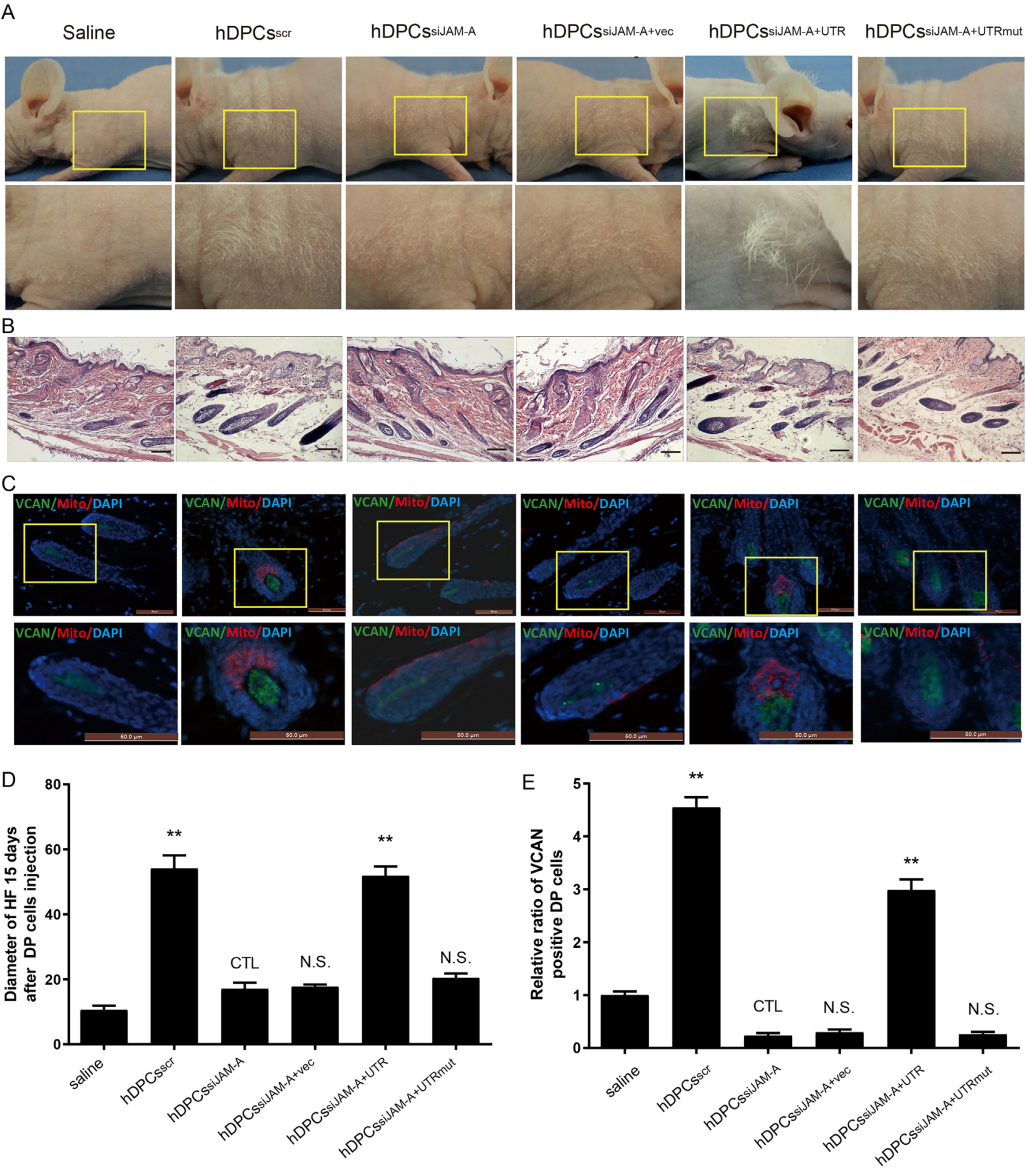
Expression of the JAM-A 3′ UTR promotes hair growth in nude mice. BALB/c-nu/nu mice were divided into six groups: saline, hDPCs^scr^, hDPCs^siJAM-A^, hDPCs^siJAM-A+vec^, hDPCs^siJAM-A+UTR^, and hDPCs^siJAM-A+UTR mut^. (**A**) Gross observation. (**B**) Histological observation. (**C**) Immunohistofluorescence observation. (**D**) Quantification of regenerated hair diameter in six groups. (**E**) Quantification of VCAN expression in hair follicles in different groups. All quantifications were done with three independent repeats. Data are represented as means ± SD. *P < 0.05, **P < 0.01. Scale bars show 50 μm.

We checked the hair regrowth after cell injection. Then, 14 days after engraftment, we found hair regrowth in hDPCs^siJAM-A+UTR^ and hDPCs^scr^ group mice (Fig. 5A). Histological analysis of the skin from these two experimental groups revealed that the HFs structure was mature, which correlated with enhanced hair growth (Fig. 5B). Hair density, hair length, and hair diameter in the six groups were all measured and compared. Statistical analysis indicated that the HF numbers and size of dermal papilla were bigger than other groups (Fig. 5C, D). These findings demonstrate that *in vivo* expression of functional JAM-A, specifically 3′ UTR, can promote and restore HF self-renewal. Subsequently, we used immunofluorescence microscopy to detect the expression of human mitochondria and VCAN in neonatal HFs (Fig. 5C, E). This experiment demonstrated that hDPCs had prominently migrated into the neonatal HFs in the nude mice: JAM-A 3′ UTR enhanced the ability of hDPCs to promote hair regrowth.

### Effect of JAM-A 3′ UTR on hair regrowth in mice with AA

**Figure 6.**
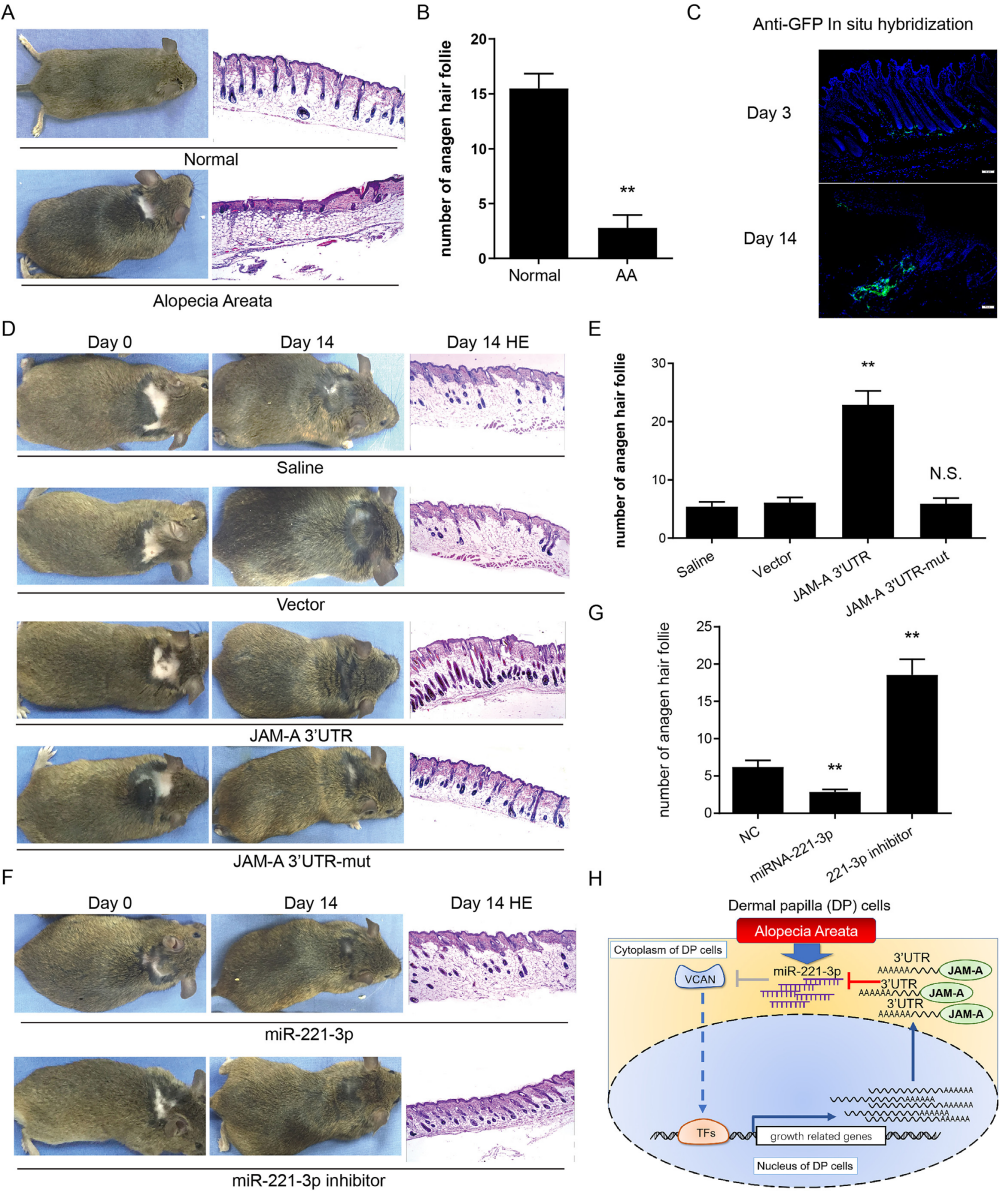
JAM-A 3′ UTR expression promotes hair regeneration in AA mice. (**A**) C3H/HeJ mice with induced AA were used to examine the decreased numbers of anagen phase hair follicle microstructures in AA skin tissue. The morphology and histology analysis and imaging were performed. (**B**) Quantifications of anagen HFs in the AA area were calculated and compared. (**C**) Representative imaging showing the integration of injected lentivirus in the HFs using anti-GFP immunofluorescence on days 3 and 14 after the injection. The results showed that injected lentivirus successfully integrated into the HFs after 14 days. (**D**) Distinct hair regeneration was observed in mice injected with JAM-A 3′ UTR Lentivirus at 14 days after engraftment (left). The histology showing the status of hair follicles in different treatment groups were shown in the right panel. (**E**) The number of anagen phase hair follicles in the JAM-A 3′ UTR injection group was significantly higher than among other groups (right). (**F**) Distinct hair regeneration was observed in mice injected with miR-221–3p mimics or inhibitor at 14 days after engraftment (left). The histology showing the status of hair follicles in different treatment groups were shown in the right panel. (**G**) Quantifications of anagen HFs in the AA area were calculated and compared. (**H**) Illustration summarizes the mechanism of how JAM-A protects VCAN expression in AA by sponging up miR-221–3p.

To explore the roles of the JAM-A 3′ UTR in hair regeneration in cases of AA, we applied adequate Imiquimod smear to the skin once or twice daily for four consecutive weeks to induce AA in 6-week-old C3H/HeJ mice (Fig. 6A).^28–30^ Imiquimod was used to induce the catagen stage,^30^ but microscopic observation showed that the HFs were in the telogen phase (Fig. 6A, B). A possible reason is C3H/HeJ model mice develop an AA-like hair phenotype spontaneously.^38^ Next, we injected JAM-A 3′ UTR lentivirus into the AA area, which was in the quiescent stage, at a concentration of 10^7^ TU per 100 μl of DMEM. 14 days after injection, the anagen HF amount in JAM-A 3′ UTR injection group was significantly higher than other experimental groups, followed by the JAM-A 3′ UTR with miR-221–3p binding site mutation group (Fig. 6C, D and E). The miR-221–3p inhibitor injection group also had a good hair regrowth effect (Fig. 6F and G).

Since AA can be seen as an organ-specific autoimmune condition characterized by T cell-mediated attacks on the hair follicle, we detected the expression of TH1 cytokines in each group after JAM-A 3′ UTR injection (Supplementary Fig. S6). A weak downward trend was seen in the JAM-A 3′ UTR injection group.

Together, these results indicate that JAM-A 3′ UTR can rescue hDPCs agglutinative growth *in vitro* and hair regeneration *in vivo*. MiR-221–3p plays a critical role in the JAM-A 3′ UTR-mediated regulation of VCAN expression and hair regeneration (Fig. 6H).

## Discussion

Given the limited number of treatments available, the need to discover new therapies for preventing hair loss and enhancing hair regrowth is urgent. This is an opportunity and a challenge for biological therapy. Replenishing DP cells with *in vitro* cultured DP cells is a feasible method to promote the telogen-to-anagen transition in the hair follicle cycle.^6^ However, DP cells will lose their hair-inducing capacity and agglutinative growth over time when they are cultured over six passages.^39^ Past studies demonstrated that cell aggregation ameliorated biological properties and hair induction ability of cultured DP cells *n*(1–3). VCAN improved DP cells self-aggregative capacity,^40^ strong VCAN expression around the matrix area could thus contribute to keep matrix cells in a poorly differentiated and proliferative phenotype.^41^ How to regulate the expression of VCAN in hair growth has not been reported yet. Using a screen for target gene prediction of abundant miRNAs in AA scalp tissue^25,42^ (GSE68801) miRNAs highly expressed in AA tissue have multiple binding sites on both genes, especially JAM-A and VCAN (Supplementary Table S4). Our previous studies had reported that JAM-A can regulate the proliferation and secretion of MSCs, promote wound repair and hair regeneration.^21,43^ So we speculate that JAM-A is likely to regulate the expression of VCAN and DP cells growth. Interestingly, we found that the non-coding region of JAM-A was at work. JAM-A 3′ UTR functions as a ceRNA to protect VCAN in self-renewing DP cells. JAM-A 3′ UTR may be a stronger stimulus to restore DP cells phenotypic marker.

Considering that miRNAs target multiple genes in the regulation of DP cells differentiation and renewal,^44^ we hypothesize that miRNA-221 maybe the bad guy that block hair regeneration. In contrast to previous models, our data led us to conclude that DP cell self-renewal is maintained by a regulatory loop in an integrated network of transcriptional and posttranscriptional miRNA/3′ UTR interactions. This regulatory loop maintains a relative balance between JAM-A/VCAN/miRNAs transcripts by suppressing the generation of DP cells in response to slight environmental changes or by initiating a rapid response to strong differentiation signals that promote DP cell differentiation or senescence.

In conclusion, we identified the JAM-A 3′ UTR as a regulator of hDPCs self-renewal, which is primarily involved in initiating the emergence of hair, especially given that its expression is substantially elevated during AA. However, despite its changes and functions in the artificial processes, we also showed that endogenous expression of the JAM-A 3′ UTR is critical for DP cell maintenance, which has strong implications for developmental biology studies of hair growth, as well as for clinical applications, going forward. Additionally, future studies will incorporate experiments examining the effects of ectopic JAM-A 3′ UTR expression on modulation of the self-renewal state of *in vitro* cultured DP cells. A comprehensive understanding of the regulatory mechanisms governing DP cells self-renewal may facilitate the development of DP cell-based therapies for AA, third-degree burns, and engineered skin tissue. Furthermore, given the widespread expression of VCAN and JAM-A, JAM-A 3′ UTR may also contribute to the regulation of genetic networks for tissue development and regeneration in other tissues than follicles, and may lead to novel therapies for many diseases.

## Supplementary Material

pbac020_Supplemental_File
