# Supplementary material for: JAM-A facilitates hair follicle regeneration in alopecia areata through functioning as ceRNA to protect VCAN expression in dermal papilla cells
Source: Precis Clin Med. 2022 Aug 17;5(3):pbac020. doi: 10.1093/pcmedi/pbac020 (PMC9486988; doi:10.1093/pcmedi/pbac020)
Supplement: pbac020_Supplemental_File [file pbac020_supplemental_file.docx]

**Supplementary Materials**

**JAM-A expression is positively correlated with** **the** **agglutinative growth of hDPCs**

In-situ hybridization results showed that both JAM-A and VCAN were mainly distributed in dermal papilla *in vivo* (Fig.S1 A). Specifically, in the cytoplasm of p3 passages hDPCs *in vitro* (Fig. S1B). We also found that primitive, early-passage hDPCs (passage 3, p3) gather in clusters when growing, which was consistent with previous reports. Concurrently, we examined alkaline phosphatase expression, because of its correlation with hDPCs capacity for induction of hair formation (Fig. S1 E). In this case, high levels of alkaline phosphatase expression were observed during the early passage, whereas in contrast, alkaline phosphatase expression and the agglutinative growth both diminished with subsequent passages of hDPCs (p7 passage) (Fig. S1 C, E).

Double immunofluorescence staining revealed colocalization and gradual downward trend of JAM-A with VCAN in different passages hDPCs (Fig. S1 D). qRT-PCR and western blot analysis results showed a marked reduction of JAM-A and VCAN in hDPCs from passages 1 to passage 7, which indicated that JAM-A expression is inversely proportional to the undifferentiated state of hDPCs (Fig. S1 F, G).

 In addition, flow cytometry (FCM) analysis further demonstrated that cell passages upregulation induced an accumulation of hDPCs cells in G1 phase (from p3 to p7 pasages), accompanied by a significant decrease in cells in S phase (Fig S1 H).  These results demonstrate that long-term in vitro culture inhibits the growth of hDPCs(p7) probably via arresting inducing G1 phase arrest. CCK-8 assays were performed to measure the proliferation inhibition of late passages hDPCs. The analysis detected a significant decrease in cell proliferation in p5 and p7 hDPCs compared with the p3 hDPCs (Fig S1 I).


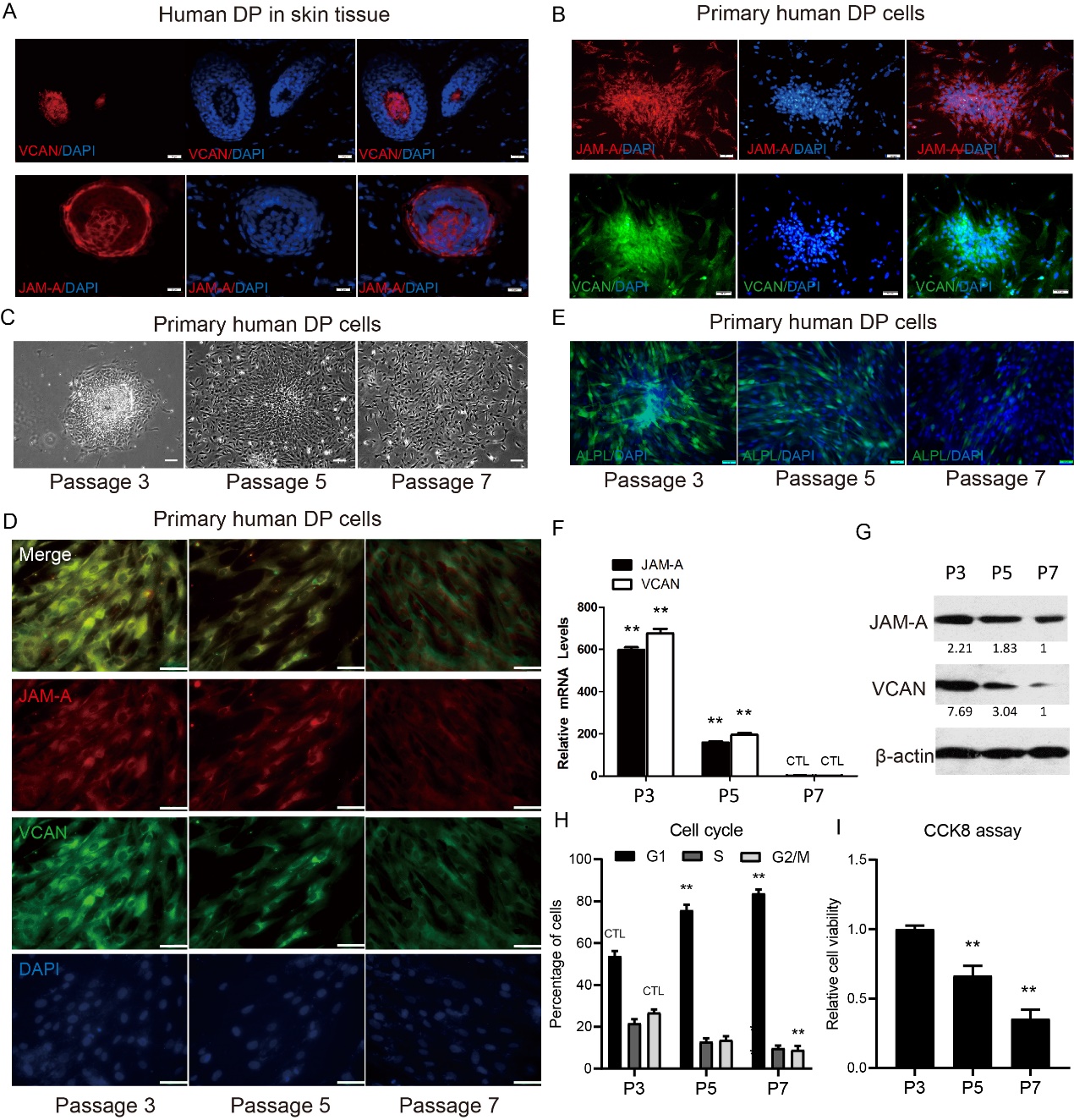


**Figure S1. VCAN and JAM-A expression is vital to maintain the agglutinative growth phenotype of hDPCs *in vitro***

1. In situ hybridization showing the localization of VCAN and JAM-A expression in human hair follicles, note that they mainly expressed in DP cells of hair follicles. (**B**) Immunofluorescence analysis of VCAN and JAM-A in primary human DP cells. (**C**) Bright field microscopy images showing the agglutinative properties of primary hDPCs between different passages in vitro. (**D**) Immunofluorescence analysis showing VCAN and JAM-A expression in primary hDPCs between different passages in vitro. All are enriched in the cytoplasm. (**E**) Immunofluorescence analysis showing AP expression in hDPCs between different passages in vitro. With the number of cell culture passages increases, the AP expression are decreases. (**F and G**) Relative RNA and protein expression of JAM-A and VCAN of hDPCs between different passages in vitro, with the extension of culture time, the expression of JAM-A and VCAN showed a downward trend. (**H**)The quantification of cell cycle analysis of hDPCs between different passages in vitro. In the 7th passage hDPCs, the G1 phase block increased, indicating that the cell proliferation ability decreased. (**I**) CCK-8 assays were conducted to determine the cell proliferation of hDPCs at early (passage 3), late (passage 7) passages. All quantifications were done with three independent repeats, and the expression of GAPDH was used as PCR internal references. Data are represented as means ± SD. *, p<0.05, **, p<0.01. Bars for 50um.


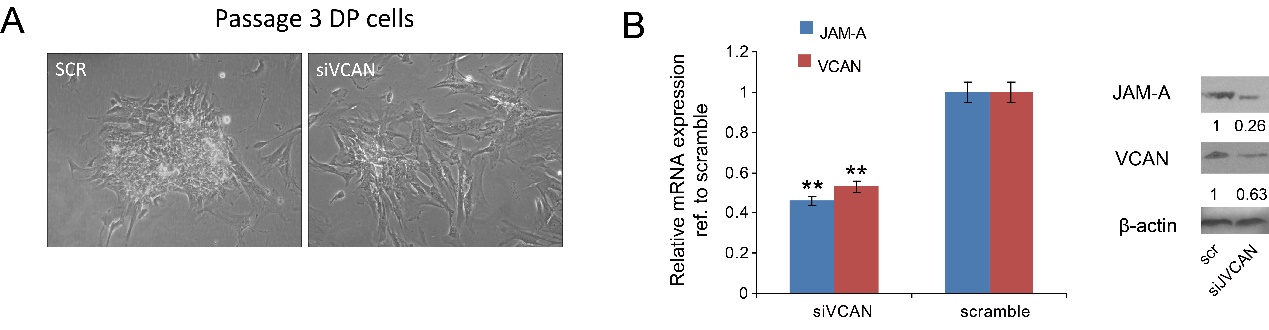
**Figure S2: hDPCs with VCAN knockdown, the phenotypic agglutinative colonies were lost**


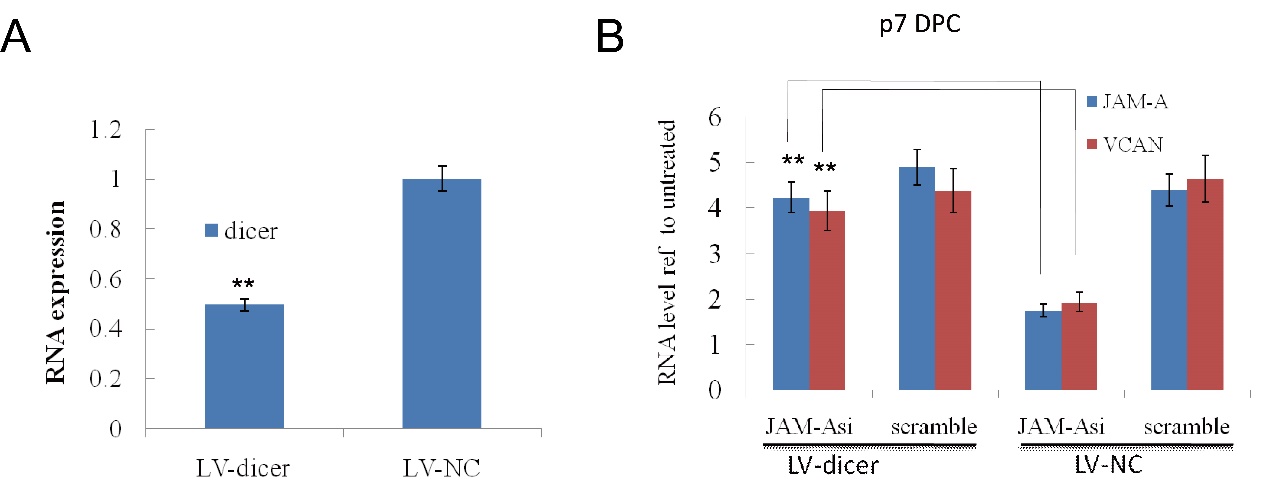


**Figure S3: p7 passages hDPCs with Dicer knockdown**


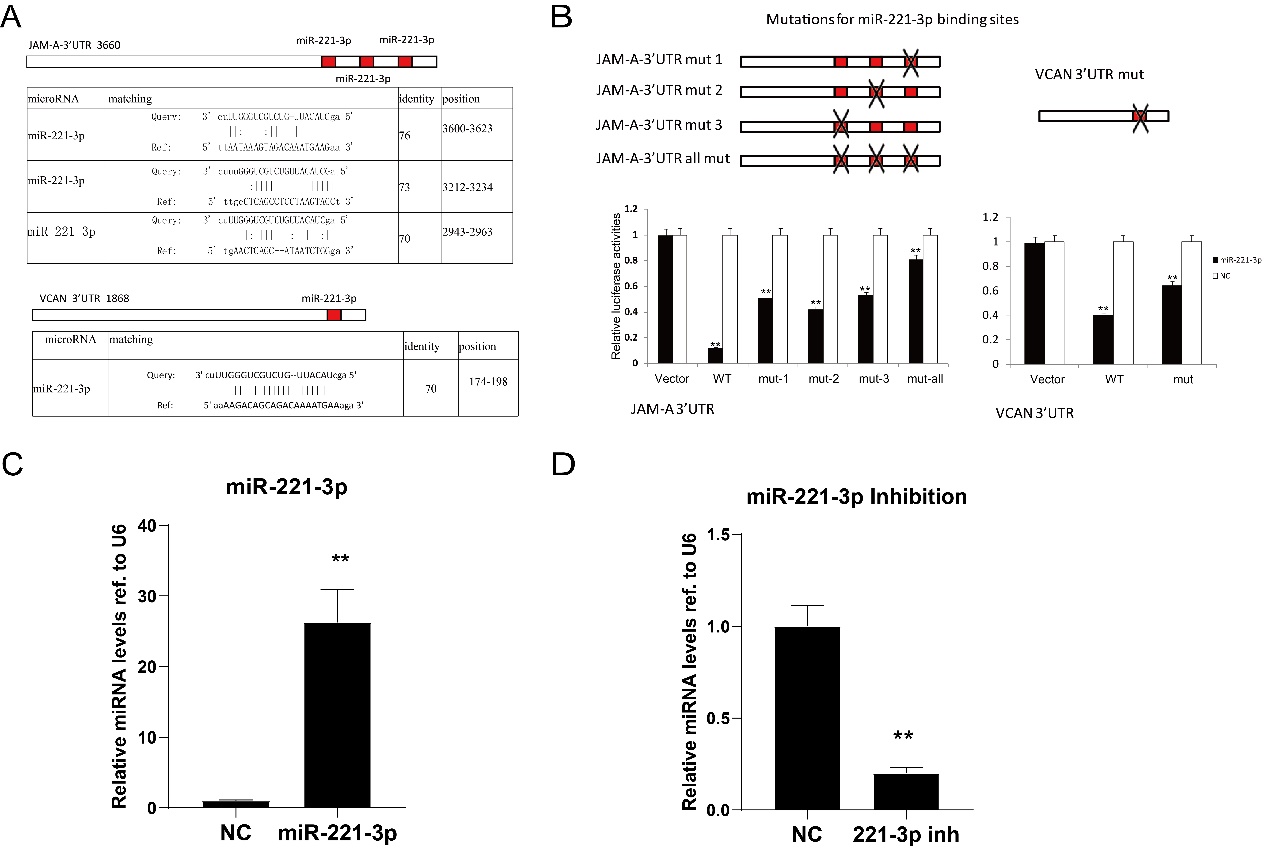


**Figure S4:** **JAM-A 3’UTR and VCAN 3’UTR with miR-221-3p binding sites mutations and miRNA**

**
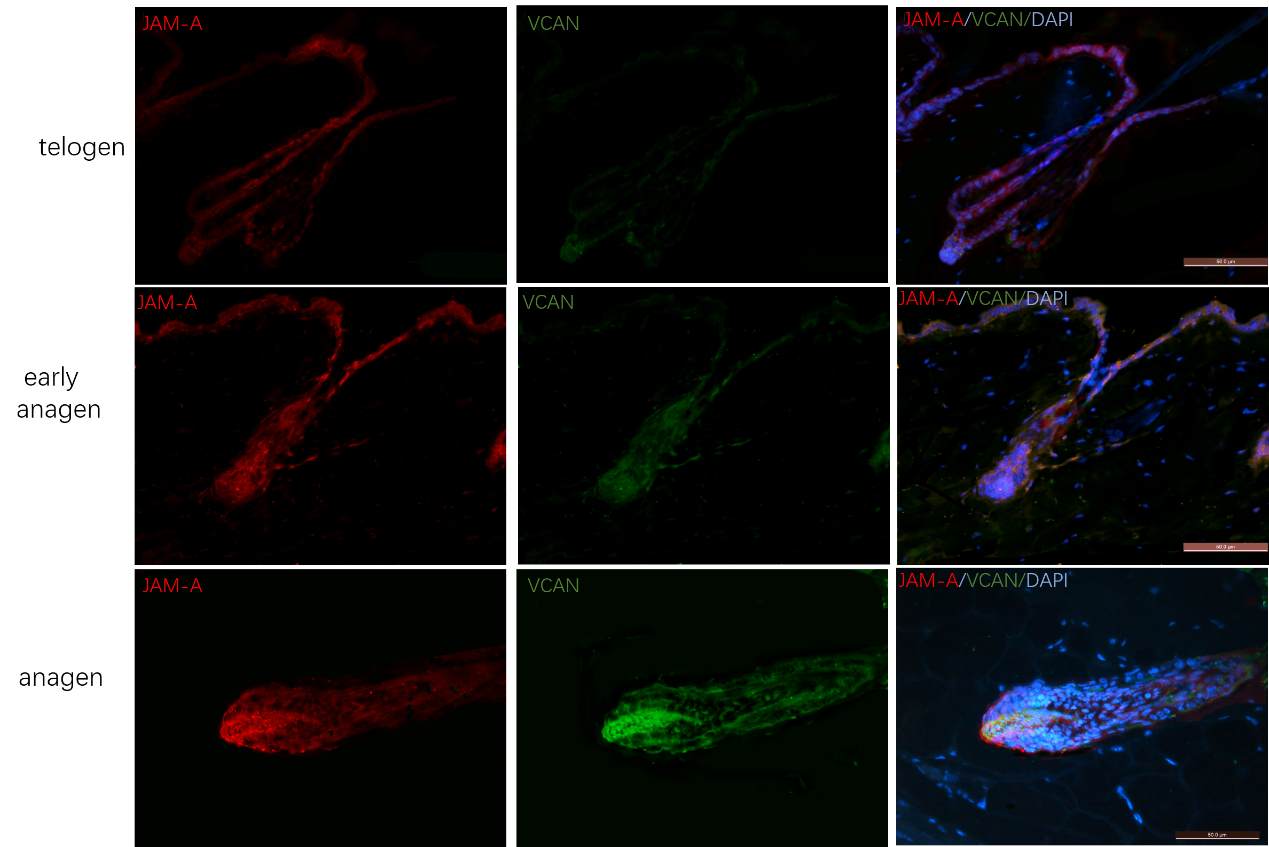
**

**Figure S5: The expression of JAM-A and VCAN in telogen and anagen hair follicle**

**Bars for 50um.**

**
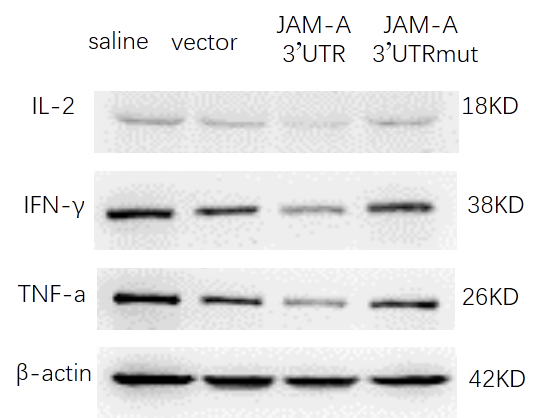
**

**Figure S6: The expressions of Th1 cytokine production, IL-2, IFN-γ, TNF-a in AA model mice injected by JAM-A 3’UTR lentivirus.**

**Table S1 The sequences of the miRNAs and siRNAs**

| Name | Sequence |
| --- | --- |
| hsa-miR-221-3p | Agcuacauugucugcuggguuuc |
| Micro RNA NC | UUCUCCGAACGUGUCACGUdTdT  ACGUGACACGUUCGGAGAAdTdT |
| hsa-inhibitor-221-3p | gaaacccagcagacaauguagcu |
| Inhibitor NC | UCUACUCUUUCUAGGAGGUUGUGA |
| Si-JAM-A | AAAGATGGGATAGTGATGCCT |
| Si-VCAN | CTTACGATGTGTATTGTTA |
| Si RNA NC | TTCTCCGAACGTGTCACGT |
| Si-Dicer | GCAGCUCUGGAUCAUAAUAUU |

**Table S2 The sequences of the primer**

| Name | Primer |
| --- | --- |
| JAM-A 3’UTR | Prime S: 5'- AGCTGAGGCAAGGGGATTTC-3'  Prime A 5'- CTGTCCGGCTCATTCCTGTT-3' |
| JAM-A CDS | Prime S: 5'- CCCTCTTGGCTTGATTTTGC-3'  Prime A 5'- TGACCTTGACTGATGGCTTC-3' |
| VCAN 3’UTR | Prime S: 5'- GCACAAAATTTCACCCTGACAT-3'  Prime A 5'- CGTTAAGGCACGGGTTCATT-3' |
| VCAN CDS | Prime S: 5'- CCACCACGCTTCCTATGTGA-3'  Prime A: 5'- TTTATGAACATCTTGGCCTTGGA-3' |
| β-actin | Prime S: 5'- CTTCCAGCCTTCCTTCCTGG-3'  Prime A: 5'- CTGTGTTGGCGTACAGGTCT-3' |

**Table S3 The miRNAs primer sequence**

| MicroRNA | Stem-loop RT primer | Forward primer |
| --- | --- | --- |
| miR-221-3p | GTCGTATCCAGTGCGAACTGTGGCGATCGGTACGGGCTACACTCGGCAATTGCACTGGATACGACgaaac | GGCAGCTACATTGTCTGCTGG |
| miR-221-5p | GTCGTATCCAGTGCGAACTGTGGCGATCGGTACGGGCTACACTCGGCAATTGCACTGGATACGACaaatc | GGCACCTGGCATACAATGTAG |
| miR-340-3p | ACACTCCAGCTGGGTTATAAAGCAATGAGA |  |
| miR-106b-3p | GTCGTATCCAGTGCGAACTGTGGCGATCGGTACGGGCTACACTCGGCAATTGCACTGGATACGACgcagcaa |  |
| miR-410-5p | CTCAACTGGTGTCGTGGAGTCGGCAATTCAGTTGAGACAGGCCA | ACACTCCAGCTGGGAATATAACACAGATG |
| URP | CTCAAGTGTCGTGGAGTCGGCAA |  |
| U6 Prime S | CGCTTCACGAATTTGCGTGTCAT |  |
| U6 Prime A | GCTTCGGCAGCACATATACTAAAAT |  |
| Premature-has-miR-221-3p Prime S | GGCATGAACCTGGCATACAATG |  |
| Premature-has-miR-221-3p Prime A | GTAGCCTGAAACCCAGCAGAC |  |
| hsa-miR-221-3p rt | GTCGTATCCAGTGCGAACTGTGGCGATCGGTACGGGCTACACTCGGCAATTGCACTGGATACGACgaaac |  |
| hsa-miR-221-5p rt | GTCGTATCCAGTGCGAACTGTGGCGATCGGTACGGGCTACACTCGGCAATTGCACTGGATACGACAAATC |  |
| U6-RT | CGCTTCACGAATTTGCGTGTCAT |  |

RT, reverse transcription; URP, universal reverse primer.

**Table S4 Predicted miRNAs targeting**

**VCAN 3’UTR, BMP-4 3’UTR, shh 3’UTR, JAM-A 3’UTR**

| MiRNA name | Positions of miRNA binding sites | | | | |
| --- | --- | --- | --- | --- | --- |
|  | VCAN | JAM-A | shh | β-catenin | BMP-4 |
| has-miR-221-3p | 2498-2522 | 4770-4793  4382-4404  4113-4133 | 286-307 | 3357-3378  417-442 488-508  2200-2224 | 126- 148  973- 994 |
| has-miR-221-5p | 1465-1487  3377-3398  1164-1185  1245-1266 | 1974-1995  4297-4318  4463-4484  212 -233  2837 -2860 | 193 - 213 | 1013-1037  2358-2379 |  |
| hsa-miR-106b-5p | 3400-3420  1599 -1618  1923 -1943 | 3400-3420  1599-1618  1923- 1943 |  | 2930-2950 |  |
| hsa-miR-106b-3p | 1284-1303  2571- 2592  1216-1240  1177-1199 | 1284-1303  2571-2592  1216-1240  1177-1199 | 283 - 305  1446-1468  570 -592 | 1070-1090  1287-1309 |  |
| has-miR-340-3p |  | 4442-4463  4391-4412  1617 -1638  21 - 43 | 903 -924 | 2962-2983  2465-2487  1689-1710  104 -125 | 490 to 511 |
| has-miR-125b-2-3p | 1542-1561  1026-1048  1619-1642 | 1249-1271  1370-1394  2523-2543  3688 -3706  2337-2358  638 - 661  1556-1577 |  | 3170-3191  24 - 43  1933-1952  1203-1224 | 1522-1542 |
| hsa-miR-125b-5p |  | 2717 -2738  2085 -2106 | 433-454  1008-1028  1039 1060 |  | 516-539  543-564 |
| hsa-miR-410-5p | 1888- 1906  2239-2263  2843-2863  807- 831 | 1888-1906  2239-2263  2843-2863  807- 831 | 820- 840  373 -396 | 1724-1744 | 998 -1019 |

**Supplement table S4 was related to figure 3.**

Table S5 The primer sequence for plasmid construction

| Name | Forward primer（5-3） | Reverse primer（5-3） |
| --- | --- | --- |
| JAM-A 3’UTR  (construction) | CCGCTCGAG GCCTGGTCGGCTCACCGCCTATC | ATAAGAATGCGGCCGC TAAAGAATTGGATATTTTTTAATGCAAATTG |
| JAM-A 3’UTR MUT1 | GGATTTTGGTGTTTCCCCTGAGACAACTTGAGTGCATAATCTGGGATAAAATGATTGA | TCAATCATTTTATCCCAGATTATGCACTCAAGTTGTCTCAGGGGAAACACCAAAATCC |
| JAM-A 3’UTR MUT2 | CACCTCCTGGGTTCAAGCAATTCTCAACGGAGTGCCTCCTAAGTAGCTGGGACTATAG | CTATAGTCCCAGCTACTTAGGAGGCACTCCGTTGAGAATTGCTTGAACCCAGGAGGTG |
| JAM-A 3’UTR MUT3 | ATGTTTCTTGGGGCCTGAGGACAGCAATTATTTCTAGACAAATGAAGAAAAACAACAAT | ATTGTTGTTTTTCTTCATTTGTCTAGAAATAATTGCTGTCCTCAGGCCCCAAGAAACAT |
| VCAN 3’UTR | ACGCGTCGAC TCCCTAAAATGGCGAACATGT | ATAAGAATGCGGCCGC GTGTAGTAAAAGAAGGATTTTAGG |
